# Short Against Long Antibiotic Therapy for Infected Orthopaedic Sites—2nd Interim Analysis of the SALATIO Trials

**DOI:** 10.3390/jcm15051695

**Published:** 2026-02-24

**Authors:** Sara Keene, Flamur Zendeli, Marc Schmid, Nathalie Kühne, Pascal R. Furrer, İlker Uçkay

**Affiliations:** 1 Unit of Clinical and Applied Research, Balgrist University Hospital, University of Zurich, 8008 Zurich, Switzerland; sarafay.keene@uzh.ch (S.K.); nathalie.kuehne@balgrist.ch (N.K.); 2 Department of Orthopedic Surgery, Balgrist University Hospital, University of Zurich, 8008 Zurich, Switzerland; flamur.zendeli@balgrist.ch (F.Z.); marc.schmid@balgrist.ch (M.S.); pascal.furrer@balgrist.ch (P.R.F.); 3 Infectiology, Balgrist University Hospital, University of Zurich, 8008 Zurich, Switzerland

**Keywords:** orthopaedic infections, short-course antibiotic therapy, antibiotic stewardship, infection recurrence, randomised clinical trial

## Abstract

**Background/Objectives:** The optimal duration of postoperative antibiotic therapy for bone and orthopaedic implant infections remains undefined. The SALATIO Trials are prospective randomised trials investigating whether shorter antibiotic courses are non-inferior to standard durations across different infection strata. This report presents the second interim analysis. **Methods:** Two unblinded non-inferiority RCTs were conducted (intention-to-treat population). Primary outcomes were remission, clinical failure, and microbiologically identical recurrence. In SALATIO 1 (material arm), participants with infected implants, retained or replaced during initial surgery, were randomised to short-course (six weeks) or long-course (twelve weeks) targeted systemic antibiotic therapy following debridement. In SALATIO 2 (non-material arm), participants undergoing implant removal or two-stage exchange were randomised to either a short-course (three weeks) or a long-course (six weeks) of antibiotic therapy. **Results:** We analysed 175 infections with a minimum follow-up period of one-year from October 2022 until July 2025: 69 (39%) in the material arm (38 short-course [55%], 31 long-course [45%]) and 106 (61%) in the non-material arm (44 short-course [42%], 62 long-course [58%]). No significant differences in clinical failure (19% overall) or microbiological recurrence (7%) were observed between treatment arms in either stratum. Multivariate analysis identified diabetes mellitus and number of debridements—but not antibiotic duration—as independent risk factors for clinical failure. Patients receiving short-course therapy experienced significantly fewer adverse events (median 0 versus 1; *p* = 0.01). Formal non-inferiority has not yet been achieved due to limited statistical power; the current analysis includes 175 of the 280 episodes (62.5%) required for the final analysis. **Conclusions:** This interim analysis suggests no disadvantage of shorter antibiotic regimens in surgically treated orthopaedic infections, whilst reducing adverse events. Patient comorbidities and surgical factors appear to be more relevant to treatment outcomes than antibiotic duration. The SALATIO Trials are ongoing and may support improved antibiotic stewardship without compromising outcomes. **Trials Registration:** NCT05499481.

## 1. Introduction

The optimal duration of postoperative systemic antibiotic therapy for bone and orthopaedic implant infections remains undefined [1]. Current guidelines recommend twelve weeks of treatment when an infected implant is retained or replaced, and six weeks following implant removal [1,2,3,4,5]. However, the rationale for precisely doubling the antibiotic duration for retained implants—regardless of debridement technique or implant characteristics—lacks scientific justification. These recommendations are largely derived from historical osteomyelitis practices and expert consensus from the previous century, established in the absence of randomised data [4]. Furthermore, the management of osteoarticular infections is inherently challenging, frequently complicated by recurrences, new infections, mechanical failures, implant loosening, gait disturbances, treatment-related adverse events, diminished quality of life, and substantial costs.

To date, these expert-driven recommendations for bone and implant infections have not undergone the same rigorous scrutiny applied to treatment protocols for other infectious diseases. Given the demonstrated success of antibiotic stewardship programmes in reducing adverse events and antimicrobial resistance across various clinical fields, there is compelling reason to pursue similar initiatives in orthopaedics, where supportive evidence remains limited [6,7,8]. Retrospective studies suggest that six weeks of treatment is as effective as prolonged courses [9,10,11,12,13], even with implant retention [9,11,13]. Additionally, these evaluations consistently fail to identify a minimum antibiotic duration threshold—irrespective of oral or parenteral antibiotic administration—below which the risk of infection recurrence rises. Concurrently, the prevalence of resistant pathogens in bone and implant infections appears to be rising worldwide [14,15]. Hence, antibiotic stewardship has been designated a priority by government agencies, regulatory bodies, professional societies, and healthcare systems alike [16]. Such stewardship is equally imperative in orthopaedic surgery, especially given that orthopaedic patients are frequently hospitalised in other departments due to advanced age, frailty, and multiple comorbidities.

At our university orthopaedic centre, we are conducting several prospective randomised trials to reduce excessive antibiotic use and improve antibiotic stewardship within the field of orthopaedic surgery [8,16,17]. The SALATIO Trials [1] extend these efforts to all bone-related infections, excluding spinal and diabetic foot infections, which are being addressed in two separate ongoing trials. For a trial of this scale and duration, interim analyses are mandatory, especially considering the theoretical risk of increased recurrence rates associated with shortened antibiotic regimens. The first interim analysis was presented at the Swiss National Congress for Orthopaedic Surgery in Lausanne (26 June 2024) [18]. The conclusion was that shorter antibiotic therapy is non-inferior compared to standard treatment regimens. This report presents the second interim analysis, performed two years after trial commencement.

### Main Hypothesis

Based on our experience and retrospective data, we hypothesised that the duration of postsurgical antibiotic therapy does not independently influence treatment outcomes in bone and implant infections. Rather, adequate surgical management is the more critical determinant. Furthermore, we posit that current standard durations are excessive for most routine cases, and that halving these durations would remain non-inferior to conventional prolonged courses in terms of clinical failure and microbiological recurrence rates. While this second interim analysis is not expected to achieve formal statistical non-inferiority within the predefined 10% margin, we anticipate no significant imbalance favouring either the short or long treatment arm. Additionally, we hypothesised that shorter antibiotic regimens will be associated with fewer antibiotic-related adverse events.

## 2. Materials and Methods

The methods employed in this interim analysis are identical to those described in our published SALATIO study protocol [1]. This section provides a condensed summary with modifications for clarity. The interim CONSORT Checklist is displayed as the Supplementary Materials.

### 2.1. Study Objectives

The objective of these interim analyses was primarily a safety and feasibility assessment in the SALATIO Trials, with a strong emphasis on the antibiotic part and its duration of administration. The SALATIO Trials aim to optimise postoperative antibiotic use in adult patients with orthopaedic infections. Two non-inferiority RCTs are conducted to evaluate whether shorter systemic antibiotic courses are non-inferior to conventional regimens. The primary outcomes are recurrent infections with the same pathogen(s) as the index episode (“microbiological recurrences”) and overall “clinical failures” (or inversely “remissions”) after the therapy for infection. The secondary outcomes were (antibiotic-related) adverse events. A third outcome would have been the assessment of surgical interventions associated with clinical failure, which we, however, skipped in this interim analysis on antibiotic treatments.

### 2.2. Definitions and Eligibility Criteria for Participants

An “orthopaedic infection” was defined as the concordant microbiological evidence of bacteria in at least two deep intraoperative tissue samples together with radiological (osteomyelitis, collections, inflammation) and/or clinical evidence of infection (pus, discharge, sinus tracts, rubor, colour, pain). Histological proof was facultative for this study. Implants were defined as any foreign material, except for allografts, transient wires, or fixator pins outside of the infected bone. The SALATIO study has two primary outcomes: “Microbiological Recurrence” and “Clinical Failure” (or inversely “Remission”), which includes “microbiological recurrences”. A microbiological recurrence was a recurrence with the same pathogen(s) after completion of antibiotic therapy. “Remission” of infection was defined as the absence of clinical, and/or radiological, and/or laboratory signs of the (former) infection after the minimal follow-up time of 12 months. Inversely, a clinical failure was any problem leading to unplanned revision surgeries, including surgeries for late-persistent infection during antibiotic therapy, non-infectious reasons (i.e., seroma, haematoma, implant failure, wound dehiscence, fractures), or a new infection with different pathogens at the same site. The reason for the existence of both primary outcomes is the academic tradition in osteoarticular infections. These infections are treated jointly by surgeons and infectiologists and published in internist or surgical journals. For surgeons the traditional outcome of interest is “Clinical Failure” or an unplanned surgical revision for any reason, whereas the only primary outcome potentially influenceable by any antibiotic treatment is the true “Microbiological Recurrence”. You can emphasise for either outcome according to your precise study interest. The SALATIO Trials investigate both. An adverse event (AE) was defined as any unexpected medical occurrence, with emphasis on antibiotic-related events. A serious adverse event (SAE) was defined as any life-threatening occurrence or one requiring rehospitalisation or significant prolongation of hospitalisation in acute-care wards.

### 2.3. Study Conduct

Eligible patients were screened according to predefined inclusion and exclusion criteria [1], and randomisation was performed at a 1:1 ratio using sealed envelopes. Table 1 resumes the inclusion and exclusion criteria, which are detailed in a prior publication [2].

- SALATIO 1, material arm: Participants with infected implants retained or replaced during initial surgery were randomised to a short-course (six weeks) or a long-course (twelve weeks) of targeted systemic antibiotic therapy (±5 days) following first debridement.
- SALATIO 2, non-material arm: Participants undergoing implant removal or two-stage exchange were randomised to a short-course (three weeks) or a long-course (six weeks) of antibiotic therapy (±5 days).

All cases required curative surgical intent from the start; patients undergoing inadequate debridement were excluded. The choice of antibiotic agent, dosage, and route of administration (oral or parenteral) was at the discretion of treating clinicians, selected from a predefined list of approved antibiotics. Antibiotic selection was guided by pathogen identification and susceptibility testing [1,19,20,21]. For staphylococcal infections with retained implants, rifampicin (450 mg twice daily for body weight ≥ 50 kg; 300 mg twice daily for <50 kg) was added to the backbone antibiotic regimen when feasible, in accordance with current guidelines for biofilm-associated infections. Oral rifampicin was initiated after wound closure to minimise resistance selection. Contraindications to rifampicin included significant drug interactions (e.g., with antiretrovirals, immunosuppressants), hepatic impairment, and rifampicin-resistant isolates [22,23,24].

Antibiotic discontinuation was determined solely by the randomised treatment duration and was not contingent on normalisation of inflammatory markers. Per protocol, antibiotics were stopped at the predetermined timepoint (±5 days) regardless of C-reactive protein (CRP), erythrocyte sedimentation rate (ESR), or white blood cell count (WBC). This approach was chosen to rigorously test the hypothesis that shorter fixed-duration regimens are non-inferior to longer courses. Laboratory values were monitored throughout treatment for safety purposes but did not influence the duration of therapy.

**Figure 1:**
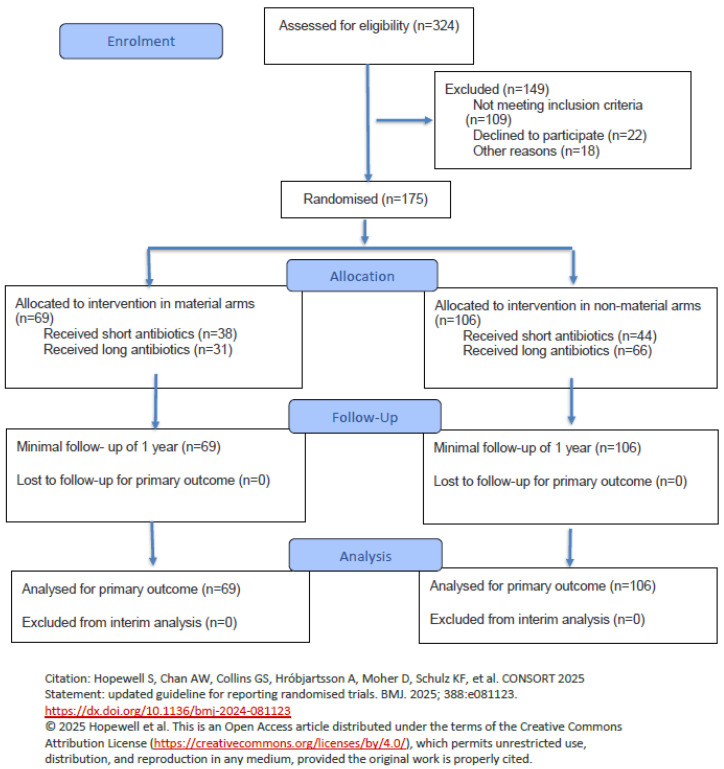
CONSORT 2025 Flow Diagram for the 2nd Interim Analysis [25].

We display the CONSORT study flow-chart for this interim analysis in Figure 1. It resumes completed infection episodes only analysed for this 2nd interim analysis and not the entire SALATIO database or other previous (interim) analyses or publications.

### 2.4. Microbiological Assessment of Infection

The microbiological assessment relied on at least two identical pathogens on intraoperative culture samples, together with the local visual signs of infection (pus, purulent liquid). The potential results of polymerase chain reaction or sonication cultures were facultative. Our specimens were processed at the same microbiological centre (Institute for Medical Microbiology, University of Zurich) based on the EUCAST criteria. The surgeons sampled a median of 5 intraoperative tissues per debridement, of which a median of 3 samples grew the same causative pathogens of infection with the same antibiotic susceptibility patterns. Sometimes, there grew an additional skin commensal in only one of the remaining enrichment broths in feeble quantity, which we regularly interpreted as contaminants.

Flow diagram of the progress through the phases of a randomised trial of two groups (that is, enrolment, intervention allocation, follow-up, and data analysis).

### 2.5. Statistical Analyses and Sample Size 

Both RCTs (material arm (SALATIO 1) and non-material arm (SALATIO 2)) are independent non-inferiority trials with a 10% margin for remission, 80% power, and two-sided α = 0.05 [1].

The assumed remission rate was set at 94% in both treatment arms. The maximum acceptable difference for the primary outcome “remission” was set at 10% (one-sided non-inferiority margin). With a two-sided α of 0.05 and 80% power, a sample size of 70 patients per treatment arm was required. Given the two strata (material and non-material arm) and two treatment durations (short-course and long-course), the total target enrolment for the final analysis is 280 infection episodes (2 × 2 × 70).

Group comparisons were performed using the Pearson χ^2^ test, Fisher’s exact test, or Wilcoxon rank-sum test, as appropriate. Futility was assessed according to established methods [26]. Multivariate logistic regression was employed to adjust for case-mix heterogeneity [27]. Formal non-inferiority was evaluated using a one-sided *p*-value threshold of <0.025 for “remission” only, as “microbiological recurrences” were too infrequent for meaningful analysis. Data were exported from REDCap^®^ to EXCEL™ and analysed using STATA™ (Version 18; StataCorp, College Station, TX, USA).

## 3. Results

### 3.1. Study Population

This second interim analysis included 175 independent infection episodes documented from October 2022 until July of 2025 with a minimum follow-up of one year and a median follow-up of 19 months (intention-to-treat population (ITT)). Six of them participated during their second infection episode. Of the 175 infections, 69 (39.4%) were in the material arm and 106 (60.6%) were in the non-material arm.

The most frequent infected material groups were infected hip arthroplasties (n = 15), knee arthroplasties and tibial plates (n = 24). The rest was distributed among other surgical teams (shoulder, foot, spine, tumour surgery, hand), involving a panoply of material infections such as screws or wires. The most common anatomical infection sites were the foot in the non-material arm, and the hip and knee in the material arm. The patient’s populations with and without infected hardware did not differ significantly according to key demographical parameters such as the biological sex, age, and antibiotic duration (all *p* > 0.05). The cohort included 64 women (37%) with a median age of 66 years and median body mass index of 25.8 kg/m^2^. Immunocompromised status was present in 38 patients (21.7%), including diabetes mellitus (n = 20; 11.4%), active malignancy (n = 5; 2.9%), and immunosuppressive medication (n = 13; 7.4%). Approximately half of the patients (n = 89; 51%) had an American Society of Anaesthesiologists (ASA) score of ≥3 points, 27% a score of 2, and 5% a score of 1 point, respectively. In terms of comorbidities, the case-mix was so heterogenous that no reasonable stratification could be made beyond the surrogate of the ASA score. As noteworthy co-morbidities beyond the above-mentioned immune suppression, we noted a severe peripheral arterial disease (n = 1), “problematic” alcohol consumption (n = 5), anaemia (n = 3), stroke (n = 2), paraplegy (n = 2), and chronic renal insufficiency (n = 2). The median C-reactive protein at admission was 91 mg/L (interquartile range [IQR], 51–161 mg/L).

The predominant pathogens were methicillin-sensitive *Staphylococcus aureus* (MSSA), *Cutibacterium acnes*, and methicillin-resistant *Staphylococcus epidermidis* (MRSE), isolated from a median of three intraoperative samples. Bacteraemia was present in only five cases (2.9%). Most patients (n = 156; 89.1%) presented with a first infection episode, whilst 19 (10.9%) had experienced one previous infection at the same site. Median sick leave duration following discharge was 54 days (IQR, 0–56).

### 3.2. Treatment

Randomisation achieved adequate balance, with 82 infections (47%) allocated to short-course and 93 (53%) to long-course antibiotic therapy (*p* = 0.08) (Table 2). Baseline characteristics did not differ significantly between treatment arms, including sex, age, body mass index, ASA score, diabetes mellitus, active malignancy, C-reactive protein level, bacteraemia, and length of hospitalisation (all *p* > 0.05) (Table 1). The median number of surgical debridements was 1 (IQR, 1–1), and there was no statistical difference between the long and short course (*p* = 0.72). The main surgical strategies for infected implants were complete removal (n = 36), partial removal (n = 27) or a mix (n = 6; e.g., using external fixation), while a possible re-osteosynthesis could occur at different timepoints. Equally, there was no difference in the use of negative-pressure wound therapy after the short antibiotic (n = 9) and the long antibiotic prescriptions (n = 5; *p* = 0.39). A total of 23 different intravenous regimens were administered for a median duration of 6 days (IQR, 2–8), followed by 36 different oral regimens for a median of 41 days. Rifampicin was added in ten cases. The most frequently prescribed agents were amoxicillin-clavulanate, levofloxacin, and clindamycin.

The length of hospital stays (median of 8 days) did not differ significantly between both randomization arms with, however, a tendency towards a shorter stay in the short arm (median 7 vs. 9 days; *p* = 0.09).

### 3.3. Outcomes 

At one year, 142 of 175 episodes (81.1%) achieved “remission”, while 33 (18.9%) experienced “clinical failure” and 13 (7.4%) had a “microbiological recurrence”. Amongst the 69 material arm infections, clinical failure occurred in 9 of 38 patients (24%) in the short-course arm (six weeks) versus 7 of 31 (23%) in the long-course arm (twelve weeks), with no significant difference between groups (*p* = 0.91). Similarly, within the 106 non-material arm infections, clinical failure rates did not differ significantly between treatment arms: 4 of 44 (9%) in the short-course arm (three weeks) versus 13 of 62 (21%) in the long-course arm (six weeks) (*p* = 0.10) (Table 3). Overall, the clinical failure rate was (15.9%) in the short-arm and (21.5%) in the long-arm group (*p* = 0.34).

In the material arm group, no significant differences were observed between patients who achieved remission and those experiencing clinical failure with respect to sex, diabetes mellitus, or number of debridement. However, patients with clinical failure were significantly older (median 78 vs. 63 years; *p* = 0.001). In the non-material arm group, the rate of diabetes mellitus was higher amongst patients with clinical failure than amongst those who had achieved remission (29% versus 6%; *p* = 0.01). Furthermore, the number of debridements was significantly higher in the clinical failure group (*p* = 0.01). We could not stratify more because of the massive underpowering of these stratified results.

### 3.4. Multivariate Adjustment

To adjust for case-mix heterogeneity and contextualise antibiotic duration within the broader clinical setting, we performed unconditional logistic regression with clinical failure as the dependent variable (Table 4). The final model included randomisation arm (antibiotic duration), age, ASA score, number of debridements, and diabetes mellitus; no significant interactions were observed amongst these covariates. For material arm infections, shorter antibiotic treatment did not increase the risk of clinical failure in either univariate or multivariate analysis (Table 3). Likewise, in the non-material arm group, antibiotic duration was not a significant predictor of outcome. However, diabetes mellitus (OR 7.6; *p* = 0.04) and number of debridements (OR 13.6; *p* = 0.002) were identified as independent risk factors for clinical failure in this group. However, the wide confidence intervals reflect limited statistical power inherent to interim analyses. Goodness-of-fit tests were non-significant for both models, indicating adequate fit.

### 3.5. Formal Non-Inferiority Assessment

Consistent with the wide confidence intervals, formal non-inferiority within the predefined 10% margin was not yet achieved in the ITT population (unidirectional *t*-test mean 0.01, 90%CI ranging from −0.18 to +0.16 for “material arm” and 0.11, 90%CI −0.01 to +0.23 for “no material arm”, respectively). A formal futility analysis was not required [26]. The independent “Data Reviewer” recommended continuation of the SALATIO Trials.

### 3.6. Adverse Events 

**Figure 2:**
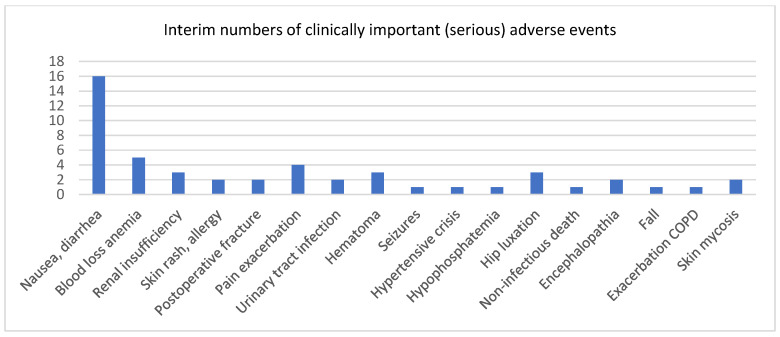
The clinically most important (serious) adverse events.

Adverse events (AEs) occurred in 92 of 175 patients (52.6%), with a median onset of 22 days following debridement. Of these, 41 (49.4%) were classified as serious adverse events (SAEs). Half of all AEs were unrelated to antibiotic therapy. The median AE duration was 23 days (IQR, 9–53). The most common adverse events were gastrointestinal symptoms (n = 16; 17.4%), including nausea and diarrhoea; haematological abnormalities (n = 5; 5.4%); dermatological reactions (n = 2; 2.2%), including rash and drug-induced exanthema; hepatotoxicity with elevated liver enzymes (n = 2; 2.2%); and nephrotoxicity (n = 3; 3.3%). The most frequently observed SAEs were death (n = 1) and wound revisions (n = 13, 14.1%). Figure 2 displays the most important interim (serious) AE at one glance. Patients in the long-course treatment arm experienced significantly more AEs than those receiving short-course therapy (median 1 versus 0 AEs, *p* = 0.01).

## 4. Discussion

This second interim analysis of the SALATIO Trials [1] yielded no unexpected findings. Consistent with the first interim analysis [18], no significant differences in remission or clinical failure were observed between short- and long-course antibiotic regimens. These findings were consistent across both strata: material arm (SALATIO 1) and non-material arm (SALATIO 2) infections. The overall clinical failure rate was high (19%), as anticipated in this population of frail patients with orthopaedic infections. Notably, this risk was not influenced by antibiotic duration. This suggests that other clinical variables are more important determinants of treatment success than antibiotic duration alone. This observation is supported by our multivariate analysis, which identified diabetes mellitus (OR 7.6; 95% CI, 1.0–55.0; *p* = 0.04) and number of debridements (OR 13.6; 95% CI, 2.6–71.0; *p* = 0.002) as independent risk factors for clinical failure in the non-material arm group, whereas antibiotic duration showed no significant effect. In the material arm group, patients with clinical failure were significantly older than those achieving remission (median 78 versus 63 years; *p* = 0.001), highlighting the role of patient-related factors over treatment duration. Adverse events occurred more frequently in long-course treatment arms (median 1 versus 0 AEs; *p* = 0.01), although no major safety concerns emerged. As expected for an interim analysis, statistical power was limited, and formal non-inferiority was not yet demonstrated. The SALATIO Trials are ongoing.

The objective of the SALATIO Trials is to reduce standard postoperative antibiotic duration by half in surgically treated orthopaedic infections, in accordance with antibiotic stewardship principles. Prior to these trials, considerable evidence suggested that such a reduction may be feasible. Retrospective case–control studies across diverse orthopaedic infections—including sacral osteomyelitis [28], long-bone osteomyelitis [29], fracture-related infections [29,30,31], prosthetic joint infections [9], and diabetic foot osteomyelitis [32] —have consistently demonstrated that longer treatment durations do not correlate with improved remission rates. Studies evaluating a standardised six-week treatment duration have shown promising results, with success rates up to 91.5% [11,33]. For sacral osteomyelitis limited to cortical bone, some authors advocate courses as short as two weeks [28]. Historically, recommended durations for bone infections ranged from four to six weeks during the twentieth century [34], with numerous case reports documenting successful outcomes with even shorter courses [35]. The current six-week standard was established in the twenty-first century, based primarily on expert consensus [4,5,29].

Our interim data aligns with these findings. The overall remission rate of 81% in our cohort is comparable to published success rates of up to 91.5% [9,10,11,30,33,36] despite halving antibiotic duration in the short-course arms. Notably, in the non-material arm, the short-course group showed a numerically lower failure rate than the long-course group (9% versus 21%), although this difference did not reach statistical significance (*p* = 0.10). This observation, while requiring confirmation in the final analysis, suggests that shorter treatment may be at least as effective as conventional durations. Preliminary RCTs have addressed similar questions in diabetic foot osteomyelitis [32] and spinal infections [37,38], with promising results supporting a reduction in antibiotic treatment duration. Studies from other institutions have reported comparable findings. Prospective randomised trials suggest that three to four weeks of systemic antibiotic therapy are sufficient following implant removal [29]. For debridement, antibiotics, and implant retention (DAIR) procedures in prosthetic joint infections, prospective data indicate that six to eight weeks of therapy may achieve outcomes equivalent to the currently recommended twelve weeks, even for virulent pathogens such as *Staphylococcus aureus* [39,40]. Only one RCT demonstrated superior outcomes with twelve-week treatment, and this benefit was limited to DAIR cases [36]. A study of fracture-related infections reported fewer adverse events with shortened antibiotic courses, without increased risk of treatment failure [41]. The SOLARIO Trial, a non-inferiority study of 500 patients with a design comparable to the SALATIO Trials, demonstrated that reducing antibiotic duration is feasible and associated with fewer AEs [42]. Further trials addressing this question have been proposed [43].

Our findings regarding adverse events further support this trend. Patients in the short-course arm experienced significantly fewer AEs (median 0 versus 1; *p* = 0.01), consistent with the SOLARIO Trial findings [42] and underscoring an important secondary benefit of reduced antibiotic exposure. Given that 52.6% of our patients experienced at least one adverse event, and half of these were antibiotic-related, the potential to reduce treatment-associated morbidity through shortened courses is clinically meaningful. In the SALATIO Trials [1] we did not control for the duration of initial parenteral therapy. This decision was based on consistent evidence from our own studies, including trials in diabetic foot osteomyelitis [32], as well as the broader literature [19,20,21], demonstrating no benefit of prolonged parenteral therapy in surgically treated, haemodynamically stable orthopaedic infections. The median duration of parenteral administration in our cohort was six days, consistent with the landmark OVIVA Trial [19]. Furthermore, our multivariate analysis identified patient comorbidities (diabetes mellitus) and surgical complexity (number of debridements) as the primary determinants of treatment failure, while antibiotic duration showed no significant effect. In the material arm, advanced age was the only factor significantly associated with clinical failure. These findings reinforce the concept that adequate surgical debridement and optimisation of patient-related factors may be more critical to treatment success than prolonged antibiotic therapy.

This study has several limitations. First, as an interim analysis, statistical power was limited, precluding definitive conclusions regarding non-inferiority. The wide confidence intervals reflect this underpowering and necessitate cautious interpretation of the findings. The current interim analysis includes 175 of the 280 infection episodes (62.5%) required for adequate statistical power to demonstrate non-inferiority. Definitive conclusions must therefore await the final analysis. Importantly, we included in this interim analysis only infection episodes that have been the complete minimal follow-up of one year (and not all included episodes). Likewise, the overall underpowering precluded further stratified analyses that would be even more underpowered. Second, randomisation imbalance was observed, with more patients allocated to the long-course arm (53% versus 47%), which may introduce bias. Third, this is a single-centre study conducted at a tertiary referral hospital specialising in orthopaedic infections, which may limit generalizability. Our patient population may differ from community hospitals in terms of infection severity, pathogen distribution, and surgical expertise. Furthermore, the predominance of foot infections in the non-material arm and prosthetic joint infections in the material arm reflects our institutional case-mix and may not be representative of other settings. However, the broad inclusion criteria and pragmatic study design enhance external validity within similar tertiary care settings. Fourth, anatomical categories are not mutually exclusive, and overlap exists. We intentionally refrained from establishing a hierarchy to include all orthopaedic infections and to avoid underpowering of the subgroup analyses. Fifth, the definition of clinical failure was broad, encompassing both infectious and non-infectious complications; this may have diluted the effect of antibiotic duration on infection-specific outcomes. Sixth, the unblinded study design may introduce detection and performance bias. However, primary outcomes were based on objective criteria rather than subjective assessments, which mitigates this concern. Seventh, fracture-fixation hardware, which is often removed after bony union, differs fundamentally from permanent reconstruction implants such as joint prostheses. In our material arm cohort, prosthetic joint infections (hip and knee) predominated, reflecting the case-mix of our tertiary centre. Subgroup analyses by implant type, and the corresponding surgical strategy, were not performed in this interim analysis due to limited sample sizes which would result in underpowered comparisons. However, the final analysis will include stratified results by implant category (prosthetic joints, fracture-fixation devices, and other implants) to address this clinically relevant question.

Finally, long-term outcomes beyond one year were not assessed; late recurrences, particularly in implant-related infections, may occur beyond this follow-up period. However, the primary purpose of our interim analysis is to identify unanticipated safety signals or futility that would warrant early trial termination. Neither concern was identified. This second interim analysis corroborates the findings of the first. The SALATIO Trials will continue, with a third interim analysis planned for late 2025, prior to the scheduled completion of follow-up in mid-2026.

## 5. Conclusions

This second interim analysis suggests no disadvantage of shorter postoperative systemic antibiotic regimens in surgically treated orthopaedic infections, while adverse events were reduced. Although formal non-inferiority within the prespecified margin cannot yet be confirmed at this stage, no safety signal indicating harm of the short-course approach was observed. The overall pattern of findings indicates that patient comorbidities and surgical factors are more relevant determinants of treatment outcome than antibiotic duration alone. These results may support improved antibiotic stewardship by reducing unnecessary antibiotic exposure without compromising clinical outcomes. While these interim results are encouraging for antibiotic stewardship efforts, definitive recommendations must await the final analysis. Clinicians should continue to follow current guidelines until non-inferiority is formally established. Despite these shortcomings, these interim results represent an important and well-executed contribution to the evolving evidence base on antibiotic stewardship in orthopaedic infections. The topic is of clear clinical relevance, the methodology is robust, and the findings are likely to stimulate further high-quality research in this field. Hence, the SALATIO Trials are ongoing, and final results will be reported in multiple publications, with stratified analyses of surgical and microbiological parameters presented separately.

## Figures and Tables

**Table 1:** Brief summary of the official SALATIO study criteria.

Inclusion Criteria	Exclusion Criteria
All adult orthopaedic bone and implant infections	Patients unable to consent (cognitively or legally)
Patient operated for infection	Conservative antibiotic treatment only
First- or second episode of the same infection	Already at least 3 episodes of the same infection
Preoperative antibiotic therapy for <5 days	Long antibiotic treatment before transfer to our centre.
Usual pyogenic bacteria	Unusual bacteria (mycobacteria, nocardia, actinomyces)
Minimal follow-up 1 year	Anticipated loss to follow-up
	Diabetic foot and vertebral osteomyelitis (in other studies)

**Table 2:** Baseline characteristics of participants by treatment arm.

	Category	n (%)	Short Treatment Arm	Long Treatment Arm	*p* Value *
Total patients		175 (100%)	82 (47%)	93 (53%)	
	Sex, female ^b^	64 (37%)	34 (53%)	30 (47%)	0.21
	Age ^a^		63 years	67 years	0.08
	Body Mass Index ^a^		25.3 kg/m^2^	26.7 kg/m^2^	0.27
	ASA score ≥ 3 points ^b^	74 (42%)	33 (45%)	41 (55%)	0.1
	Diabetes mellitus ^b^	20 (100%)	6 (30%)	14 (70%)	0.11
	Active malignancy ^b^	5 (100%)	3 (60%)	2 (40%)	0.55
	C-reactive protein level ^a^		62.2 mg/L	108.7 mg/L	0.51
	Bacteraemia ^b^	5 (100%)	1	4	0.22
	Length of hospitalisation ^a^		7 days	9.5 days	0.09
Infection type	Material arm ^b^	69 (39%)	38 (55%)	31 (45%)	0.08
	Non-Material arm ^b^	106 (61%)	44 (42%)	62 (58%)	

^a^ Values are expressed as the median. The Mann–Whitney U test was used to analyse the differences between the short treatment arm and the long treatment arm. ^b^ Values are expressed as absolute numbers with percentages in parentheses. The Fisher exact or Chi-square test was used to analyse the difference between the short treatment arm and the long treatment arm. * A *p* value < 0.05 was considered significant.

**Table 3:** Baseline demographic and clinical characteristics by treatment allocation.

Material Arm (SALATIO 1)	Remission	Clinical Failure +	*p*-Value *	Non-Material Arm (SALATIO 2)	Remission	Clinical Failure +	*p*-Value *
n = 69 (100%)	n = 53 (77%)	n = 16 (23%)		n = 106 (100%)	n = 89 (84%)	n = 17 (16%)	
Female sex ^b^	18 (34%)	7 (44%)	0.48	Female sex ^b^	33 (37%)	6 (35%)	0.89
Age ^a^	63 years	78 years	***0.001***	Age ^a^	64 years	63 years	0.24
Diabetes mellitus ^b^ ASA score ^a^	8 (15%) 3	2 (13%) 3	0.80 0.33	Diabetes mellitus ^b^ ASA score ^a^	5 (6%) 3	5 (29%) 3	***0.01*** *0.98*
Number of debridement ^a^	1 intervention	1 intervention	0.08	Number of debridement ^a^	1 intervention	1 intervention	***0.0004***
Six weeks of antibiotic therapy ^b^ (n = 38) (100%)	29 (76%)	9 (24%)	0.91	Three weeks of antibiotic therapy ^b^ (n = 44) (100%)	40 (91%)	4 (9%)	0.10
Twelve weeks of antibiotic therapy ^b^ (n = 31) (100%)	24 (77%)	7 (23%)		Six weeks of antibiotic therapy ^b^ (n = 62) (100%)	49 (79%)	13 (21%)	

^a^ Values are expressed as the median. The Mann–Whitney U test was used to analyse the differences between the groups. ^b^ Values are expressed as absolute numbers with percentages in parentheses. The Fisher exact or Chi-square-test was used to analyse the difference between the groups. * A *p* value  < 0.05 was considered significant. Significant results are indicated in bold and italic. + Clinical failure = any unplanned clinical condition requiring re-hospitalisation (including revision) such as new infection, haematoma, wound dehiscence, seroma, implant-loosening, fracture, segment degeneration.

**Table 4:** Univariate and multivariate predictors of clinical failure.

Material Arm Infections	Univariate			Multivariate		
**n = 69**	**Odds Ratio**	**95%CI**	***p*** -Value *	**Odds Ratio**	**95%CI**	***p*** -Value *
Age	1.1	1.0–1.2	0.01	-		
Diabetes mellitus	0.8	0.2–4.2	0.8	0.6	0.1–3.6	0.58
° ASA score	1.5	0.7–3.3	0.36	-		
Short (6-week) antibiotic therapy	1.1	0.3–3.3	0.91	1.2	0.3–4.4	0.8
Number of debridement	2.9	0.9–9.3	0.07	3.2	0.9–11.3	0.08
**Non-material arm infections**	**Univariate**			**Multivariate**		
**n = 106**	**Odds Ratio**	**95%CI**	***p*** -Value *	**Odds Ratio**	**95%CI**	***p*** -Value *
Age	1.0	0.9–1.0	0.25	-		
Diabetes mellitus	***7.0***	***1.8–27.8***	***0.01***	***7.6***	***1.0–55.0***	***0.04***
° ASA score	1.2	0.3–10.2	0.67	-		
Short (6-week) antibiotic therapy	0.4	0.1–1.2	0.11	0.1	0.1–1.6	0.11
Number of debridement	***3.9***	***1.6–9.7***	***0.003***	***13.6***	***2.6–71.0***	***0.002***

* A *p*-value < 0.05 was considered significant. Statistically significant results are displayed in bold and italic. “-” = not included in the multivariate model due to interaction or few cases. ° ASA = American Society of Anaesthesiologists.

## Data Availability

We may provide anonymized key elements of the datasets upon reasonable scientific request.

## Supplementary Materials

The following supporting information can be downloaded at https://www.mdpi.com/article/10.3390/jcm15051695/s1, File S1: CONSORT_2025 Checklist.

## Abbreviations

The following abbreviations are used in this manuscript:

SALATIO	Short Against Long Antibiotic Therapy for Infected Orthopaedic Sites
RCT	Randomised Controlled Trial
AE	Adverse event
DAIR	Debridement, antibiotics, implant retention
SAE	Serious adverse event
MSSA	Methicillin-sensitive Staphylococcus aureus
MRSE	Methicillin-resistant Staphylococcus epidermidis
